# Fabrication of Poly(vinyl alcohol)/Chitosan Composite Films Strengthened with Titanium Dioxide and Polyphosphonate Additives for Packaging Applications

**DOI:** 10.3390/gels8080474

**Published:** 2022-07-28

**Authors:** Tăchiță Vlad-Bubulac, Corneliu Hamciuc, Cristina Mihaela Rîmbu, Magdalena Aflori, Maria Butnaru, Alin Alexandru Enache, Diana Serbezeanu

**Affiliations:** 1 Department of Polycondensation and Thermally Stable Polymers, “Petru Poni” Institute of Macromolecular Chemistry, 41A, Grigore Ghica Voda Alley, 700487 Iasi, Romania; chamciuc@icmpp.ro (C.H.); maflori@icmpp.ro (M.A.); diana.serbezeanu@icmpp.ro (D.S.); 2 Department of Public Health, Faculty of Veterinary Medicine “Ion Ionescu de la Brad”, University of Agricultural Sciences and Veterinary Medicine, 8, Mihail Sadoveanu Alley, 707027 Iasi, Romania; crimbu@yahoo.com; 3 Department of Natural Polymers, Bioactive and Biocompatible Materials, “Grigore T. Popa” University of Medicine and Pharmacy, 700115 Iasi, Romania; mariabutnaru@yahoo.com; 4 S.C. Apel Laser S.R.L., 25, Vanatorilor Street, Mogosoaia, 077135 Ilfov, Romania; alin.enache@apellaser.ro

**Keywords:** poly(vinyl alcohol), chitosan, titanium dioxide, polyphosphonate, casting from solution, xerogel composite film

## Abstract

Eco-innovation through the development of intelligent materials for food packaging is evolving, and it still has huge potential to improve food product safety, quality, and control. The design of such materials by the combination of biodegradable semi-synthetic polymers with natural ones and with some additives, which may improve certain functionalities in the targeted material, is continuing to attract attention of researchers. To fabricate composite films via casting from solution, followed by drying in atmospheric conditions, certain mass ratios of poly(vinyl alcohol) and chitosan were used as polymeric matrix, whereas TiO_2_ nanoparticles and a polyphosphonate were used as reinforcing additives. The structural confirmation, surface properties, swelling behavior, and morphology of the xerogel composite films have been studied. The results confirmed the presence of all ingredients in the prepared fabrics, the contact angle of the formulation containing poly(vinyl alcohol), chitosan, and titanium dioxide in its composition exhibited the smallest value (87.67°), whereas the profilometry and scanning electron microscopy enlightened the good dispersion of the ingredients and the quality of all the composite films. Antimicrobial assay established successful antimicrobial potential of the poly(vinyl alcoohol)/chitosan-reinforced composites films against *Staphylococcus aureus*, *Methicillin-resistant* *Staphylococcus aureus* (MRSA), *Escherichia coli*, *Pseudomonas aeruginosa*, and *Candida albicans*. Cytotoxicity tests have revealed that the studied films are non-toxic, presented good compatibility, and they are attractive candidates for packaging applications.

## 1. Introduction

Plastic packaging continues, in recent years, to be the most significant industrial use in the world (representing, on average, 30% of the total) [1,2,3]. Particularly, polymers, like polyethylene terephthalate, low-density polyethylene, polystyrene, polypropylene, and high-density polyethylene, were widely used as single-use packaging materials in the food and beverage industry because of their mechanical properties that could provide effective barriers to oxygen and carbon dioxide as well as their relative affordability and ease of availability [4,5,6,7,8]. Nevertheless, when plastic packaging based on the abovementioned polymers reaches the end life of its use, a significant amount frequently eludes formal collection and recycling processes and eventually leaks away, damaging the worldwide environment [9,10,11].

According to estimates, the increase in population growth will demand a 50% increase in global food supplies by the year 2050 [12]. Therefore, it is necessary to incorporate eco-innovations in primary packaging that can lessen the impact of packages on the environment and simultaneously maintain food quality and safety [13]. Additionally, more and more efforts are being put into creating bio-products for eco-innovations in food packaging, like biodegradable and compostable polymers [14,15].

The primary choice for packaging is represented by natural polymers, which should be researched and used extensively in the near future. The second-most prevalent polysaccharide in the world, chitosan, CS, is a biocompatible, biodegradable, and nontoxic substance that has proven to be a good candidate for use in packaging films. Structural versatility, attractive barrier properties, excellent film-forming and coating capabilities, as well as an innate antibacterial capability, have been proven for this biopolymer. As a result, several films made from CS have been produced and used in the food packaging sector [16,17,18,19]. Pure CS, however, is only soluble in acidic environments and has poor mechanical and thermal properties [20]. A unique bio-composite material with brand-new or improved properties could be produced by mixing biopolymers with existing polymeric matrices, natural or semi-synthetic, to meet specific needs. Such combinations of CS with starch, pectin, alginate, poly lactic acid (PLA), poly(vinyl alcohol) (PVA), gelatin, etc. have been reported to date [21,22,23,24,25].

Blends of PVA with CS, which combine a natural biomacromolecule and an easily biodegradable synthetic polymer, are among the most attractive bicomponent systems [26]. PVA is a thermoplastic synthetic polymer created from the hydrolysis of poly(vinyl acetate), unlike many other synthetic polymers. Due to PVA’s distinct characteristics, which include high mechanical properties, chemical and thermal stability, non-toxicity, film-forming skills, and low manufacturing costs, its uses have increased over the past ten years. In addition to biodegradable goods like backing rolls, adhesives, coatings, and surfactants, PVA is utilized in a number of different industries, including those that deal with textiles, paper, and food packaging [27]. PVA, like other synthetic polymers, has applications in biology and medicine in addition to technical ones, and this has led to it being one of the main research areas for polymer scientists. Additionally, in recent years PVA have been studied for various smart applications such as shape memory hydrogels with improved viscoelasticity for printable applications [28], bio-based sensors and antimicrobial films [29], nanofibrous metallochromic sensors for colorimetric selective detection of ferric ions [30], etc.

It has also been shown that adding fillers to this PVA/CS bicomponent systems can be supplementary reinforcement, creating new composites with enhanced physical features, like water resistance, without sacrificing biodegradability. Because of their simplicity of processing, low cost, and superior synergistic characteristics, nanomaterials have been produced and employed in a wide variety of fields, including foods, medicines, and cosmetics. Incorporating nanosized compounds like nano-ZnO, nano-TiO_2_, and nano-silica into the polymers has been proven in some studies to help improve the characteristics of the polymers [31,32,33]. In our recent paper, synergistic effects of the presence of silica nanoparticles and a polyphosphonate mixed into PVA matrix have been discussed [34].

TiO_2_ is a versatile and chemically inert mineral with various pertinent uses (food, pharmaceutical, biomedical, antibacterial agent, environmental, and clean energy) [34]. Despite the fact that its use as a food additive has recently been questioned and even banned in the European Union, TiO_2′_s physicochemical, mechanical, and photocatalytic qualities, as well as its reactivity and thermal stability, low cost, secure manufacturing, and biocompatibility, all contribute to TiO_2′_s widespread application [35]. However, the capacity of nano-TiO_2_ to aggregate is one of its main drawbacks. According to certain reports, the interaction of nano-TiO_2_ with biopolymers including starch, gums, and chitosan can aid to lessen the spontaneous agglomeration of TiO_2_, improving the functional aspects of the composite [36]. The addition of nanoparticles to the polymer matrix has two benefits: first, it strengthens and functionalizes the matrix by improving its dispersibility, and second, it gives the mixture antibacterial capabilities [37].

In composite materials, PVA is frequently utilized as a polymer matrix, and CS is frequently employed as a reinforcing agent in an effort to broaden the application of PVA in the field of high-strength materials [26,38]. Thus, the co-existence of the hydroxyl groups in both PVA and CS, doubled by the presence of amino groups in CS, can produce intermolecular interactions, which may result in an improvement of performances of PVA composites. The use of polyphosphonate containing sulfur and phosphorus in the main chain and in the side chain in phosphaphenanthrene-type heterocycles is expected to induce thermal stability in the composite, then, to synergistically bring its own antimicrobial contribution to the composite, as was described in few studies in the recent past [39].

**Figure 1:**
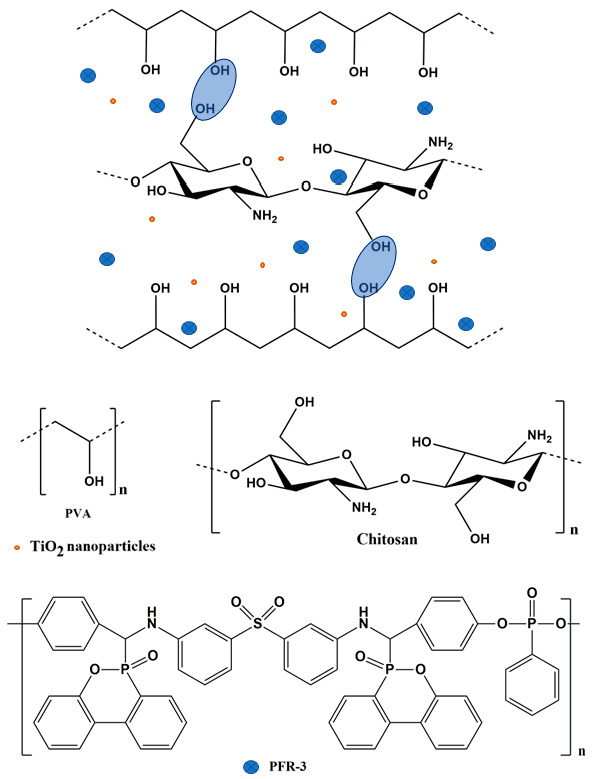
Schematic representation of PVA/CS/TiO_2_/polyphosphonate composites.

In the present study, optimized solutions of PVA/CS, PVA/CS/TiO_2_, or PVA/CS/TiO_2_/polyphosphonate have been used to obtain xerogels composite films via casting from solution method, followed by drying in ambient conditions. Schematic representation of the composites is presented in Figure 1.

The effect of all the ingredients on the structure-properties relation of the developed xerogel composite films was determined and discussed. Physical, chemical, morphological properties, surface properties, antimicrobial activity, and cytocompatibility of the matrices were studied by means of FTIR, SEM, swelling behavior, profilometry, contact angle measurements, antimicrobial, and cytotoxicity assays.

## 2. Results and Discussion

### 2.1. Preparation and Structural Characterization

Binary PVA/CS films and the composite PVA/CS films containing TiO_2_ or TiO_2_ with polyphosphonate, PFR-3, have been obtained by the casting from solution procedure. The details of this procedure are presented in the Materials and Methods section. The composition of the composite polymer matrices expressed in mass ratio for all the ingredients utilized in the preparation and the codes of the as-prepared composites are listed in Table 1.

**Figure 2:**
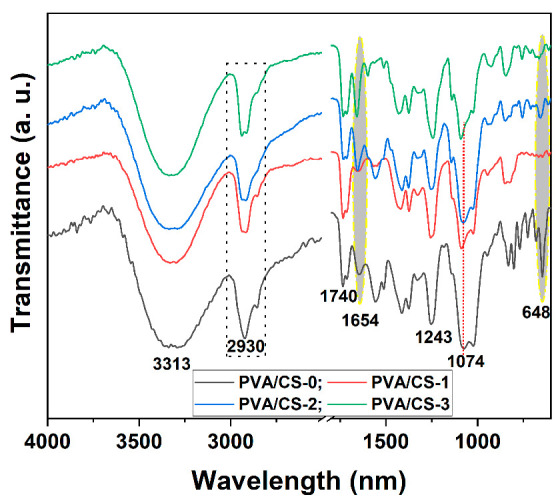
Fourier-transform infrared (FTIR) spectra for PVA/CS composites.

The chemical structure of the products was introspected by FTIR spectroscopy. Figure 2 presents the FTIR spectra of the binary PVA/CS-0 sample and of the PVA/CS composite films. In the spectrum of the PVA/CS-0 sample, the characteristic strong and wide band appeared at approximately 3320 cm^−1^ due to hydroxyl stretching vibration.

Common characteristic absorption bands were found also at about 2930 cm^−1^ with a shoulder at 2860 cm^−1^ due to asymmetric and symmetric stretching vibrations of C–H, at 1414 cm^−1^ (δ_C_–_H_), 1378 cm^−1^ (ω_C_–_H_), 1243 cm^−1^ (ω_C_–_H_), 1074 cm^−1^, 1023 cm^−1^ (ν_C_–_O_), and 830 cm^−1^ (ρ_CH2_) [40]. Due to C=O and C–O–C units, respectively, characteristic absorption bands for the non-hydrolyzed ester groups−O–CO–CH_3_ were observed at 1735 cm^−1^ and 1245 cm^−1^. The characteristic bands for CS could be also assigned in the FTIR spectra of the samples, at 1556 cm^−1^ (band characteristic for amide II, ν_N_–_H_), 2920 cm^−1^ (ν_C_–_H_), and approximately 3300 cm^−1^ (wide band with νNH vibration overlapping with νO–H of polyvinyl alcohol). The characteristic band appearing at 1557 cm^−1^ assigned for δ_NH_ (amide II) vibration of the NH_2_ group, and the band observable at 1654 cm^−1^ assigned for amide I (ν_C=O_) of O=C–NHR groups, were revealed to be diminished and slightly shifted in the FTIR spectra of the ternary and quaternary composite films (samples PVA/CS-2 and PVA/CS-3), indicating some interactions between CS and PVA that could be catalyzed by the presence of the additives in the samples. Additionally, the band appearing enlarged at 846 cm^−1^ in the multi-component composites (samples PVA/CS-1, PVA/CS-2, and PVA/CS-3) is the indicative of occurrence of hydrogen bonds formed between OH groups of the ingredients present in the composites.

From the FTIR spectra of the PVA/CS-2 and PVA/CS-3 samples, characteristic absorption bands at 1470 cm^−1^, 1210 cm^−1^ (appearing as small shoulders), and 1140 cm^−1^ (appearing as a distinctive peak), were assigned to stretching vibration of the P–Ar, P–O and P–O–C, respectively. Another distinctive band confirming the presence of the PFR-3 additive into the composition of the PVA/CS-2 and PVA/CS-3 samples could be observed as a sharp peak near 755 cm^−1^, which is attributed to the P–O–Ph group.

### 2.2. Surface Characteristics of the PVA/CS Composite Films

Due to their outstanding biocompatibility, resistance to bodily fluids, mechanical qualities, anticorrosive capability, and flexibility, titanium-based composites are preferred for use in biomedical applications [41,42]. However, their properties depend on the surface, which is related with the mixing capacity of nanomaterials with titanium oxide [43]. Accordingly, it has been suggested that the interaction between titanium oxide nanoparticles (TiO_2_) and biopolymers (starch, gums, and chitosan) can aid in reducing the spontaneous agglomeration of nanoparticles, thereby enhancing the functional qualities of the composite [36].

**Figure 3:**
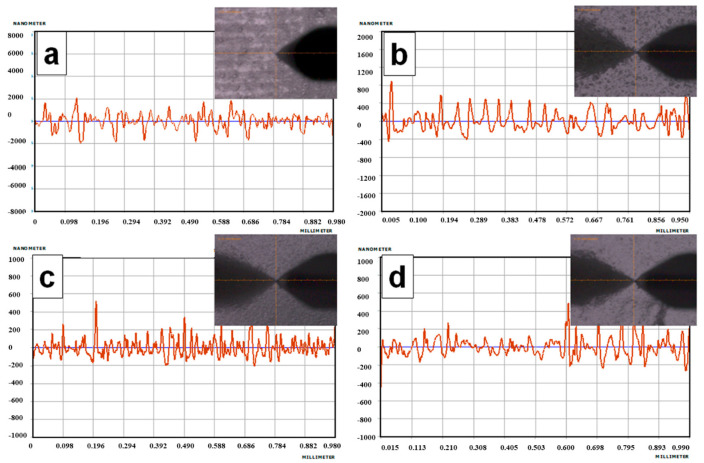
Microscopic images of the composite films and histograms generated by Alpha-Step D-500 Stilus profilometer (**a**) PVA/CS-0; (**b**) PVA/CS-1; (**c**) PVA/CS-2; (**d**) PVA/CS-3.

PVA/CS composite films were analyzed using a profilometer. Microscopic images of the composite films were taken (histograms generated by the profilometer) and are presented in Figure 3, and the average roughness values were calculated (Table 2). According to the histograms and the microscopic images, it has been revealed that the addition of titanium dioxide to the polymeric matrix based on chitosan and PVA resulted in the production of composite films with a homogeneous composition and a surface devoid of cracks. The average roughness, Ra, representing the arithmetic mean of the highs and lows profiles, was in the interval of 65.5–570.3 nm. The surface roughness parameters decreased when introducing the PFR-3 in the PVA/CS matrix, according to the Ra value (Table 2), suggesting its contribution to the homogeneity and quality of the multi-component composites.

**Figure 4:**
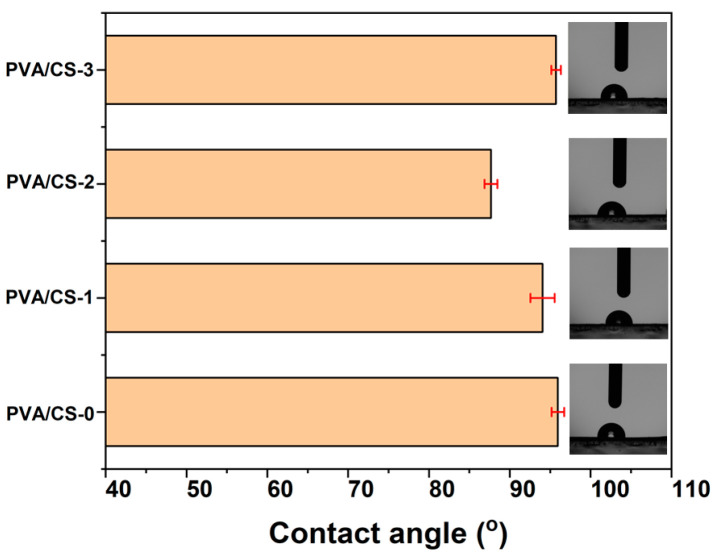
Contact angle of water on PVA/CS samples.

The wetting characteristics, such as work of adhesion (Wa), the total solid surface free energy (γ_SV_), solid-liquid interfacial tension (γ_SL_), etc., of the PVA/CS samples with respect to the W and EG were investigated. A summary of all contact angle study parameters is provided. Table 2 provides a summary of all the contact angle investigations’ parameters. W and EG were used as the test liquids, whereas PVA samples were examined using contact angle measurements at room temperature. Figure 4 displays the results of measuring the contact angle for the PVA sample in W.

For water, the PVA/CS-0 sample exhibited a contact angle of 95.38° while by adding 0.16 g TiO_2_ a slight decrease of the contact angle to 94.05° was observed in the case of the sample PVA/CS-1; a decrease was also observed in the roughness of the respective sample (Table 2). The same behavior, in terms of contact angle and roughness decreases, has been noticed when adding 0.2172 g of polyphosphonate PFR-3, which resulted in a further decrease of the contact angle as a result of the presence of phenyl-phosphonate groups within the macromolecule of the polymeric additive that could influence the molecular hydrogen bonding; these results are consistent with the FTIR findings. Thus, in the case of PVA/CS-2, the contact angle value decreased to 87.67° due to the orientation of macromolecules and the reorganization of the polar groups from the surface of the composite sample. After continuing to add the PFR-3 additive (0.4344), the hydrophobic character was preserved as a result of the reduced concentration of OH groups, whereas the phenyl-phosphonate group’s impact was disrupted by the presence of bulky, rigid phosphaphenanthrene groups.

When polyphosphonate PFR-3 was added into the PVA matrix, the work of adhesion (Wa) increased (PVA/CS-2 sample). Therefore, an increase in the work of adhesion may have resulted from the polyphosphonate’s effective dispersion in the PVA/CS matrix. For the PVA/CS-0 sample, γ_SV_ was 22.54 mN/m and increased up to 38.92 mN/m in the PVA/CS-2 sample, whereas the sample containing TiO_2_ nanoparticles presented the highest value of 49.84 mN/m, suggesting the different nature of the forces interacting on the surface of the different compositions. Table 2 revealed that samples PVA/CS-1 and PVA/CS-2 show lower polar surface parameters γ^P^ SV due to the presence of nano powders in the polymeric matrices. After continuing to add PFR-3 additive, a decrease of the Wa value and an increase of the polar surface parameters γ^P^ SV were achieved, probably due to the increase in the hydrophobicity of the surface of the sample PVA/CS-3. Additionally, from Table 2 it can be observed that the dispersive component values for the PVA/CS samples are greater than the polar component values, which leads to the conclusion that there are more hydrophobic groups than hydrophilic ones on the surface of the PVA/CS films.

### 2.3. Swelling Behavior and Morphology of PVA/CS Composite Films

**Figure 5:**
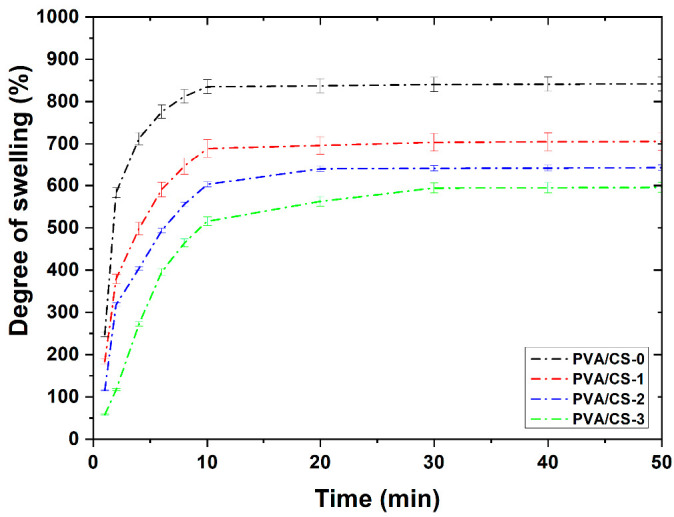
Swelling degree of PVA/CS composites.

The swelling behavior of the samples PVA/CS revealed that the PVA/CS-0 matrix without TiO_2_ and PFR-3 additive exhibited the highest maximum swelling degree achieved at equilibrium, 834% (Figure 5). For the sample containing TiO_2_, the maximum swelling degree achieved at equilibrium was 688%, whereas introducing the polyphosphonate additive into the systems further decreased the swelling behavior of the composite in the series, with the swelling degree achieved at equilibrium being 639% and 593% in the case of the PVA/CS-2 and PVA/CS-3 sample, respectively. Another observation relates to a higher initial water uptake in the first 10 min when the samples PVA/CS-0 and PVA/CS-1 reached the maximum swelling degree, whereas in the case of the samples PVA/CS-2 and PVA/CS-3, the maximum swelling degree was achieved after approximately 30 min.

**Figure 6:**
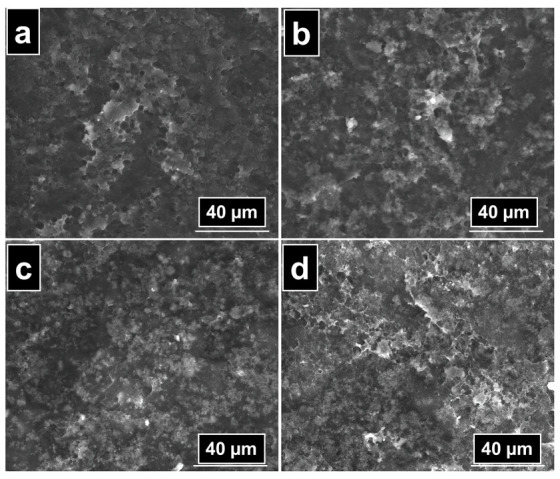
SEM images of PVA/CS-0 (**a**), PVA/CS-1 (**b**), PVA/CS-2 (**c**), and PVA/CS-3 (**d**) composite films.

The morphology of PVA/CS composite films was investigated by scanning electron microscopy. Microphotographs for PVA/CS-0 in comparison with the samples containing additive are presented in Figure 6. The morphology of the PVA/CS-0 sample appeared uniform and smooth, presenting a porous behavior. The aspect of the main polymeric matrix was also preserved in the samples containing either titanium dioxide nanoparticles or both TiO_2_ and PFR-3 additive in their structure. A slight increase in the porosity could be observed in the sample PVA/CS-3 as the content of polyphosphonate PFR-3 increased.

### 2.4. In Vitro Evaluation of Antimicrobial Potential of the PVA/CS Composite Films

Due to the chitosan and the specific conditions of the culture medium and incubation parameters (37 °C/24 h), the tested matrices changed their disc-like shape and/or lost contact with the microbial culture, leading to a limitation in the interpretation of the antimicrobial activity.

However, qualitative evaluation of the antimicrobial potential of the PVA/CS composites films against Staphylococcus aureus ATCC 25923, *Methicillin-resistant Staphylococcus aureus* (MRSA) ATCC 43300, Escherichia coli ATCC 25922, Pseudomonas aeruginosa ATCC 27853, and Candida albicans ATCC 90028 (Table 3) was successful. Samples PVA/CS-2 and PVA/CS-3 contain both TiO_2_ and PFR-3 additive in their structure. The presence of TiO_2_ at a similar concentration as in PVA/CS-1 reflects the antimicrobial nature of the matrix, which was highlighted in PVA/CS-2 against Candida albicans and in PVA/CS-3 against Staphylococcus aureus, Pseudomonas aeruginosa, and Candida albicans. At the same time, inhibition of antimicrobial activity was observed against MRSA (matrices PVA/CS-2 and PVA/CS-3), Escherichia coli (matrices PVA/CS-2 and PVA/CS-3), Pseudomonas aeruginosa (PVA/CS-2), and Staphylococcus aureus (PVA/CS-2).

Comparing the results obtained for the PVA/CS-0 film (the sample containing only PVA and chitosan) and the functionalized composite films (PVA/CS-1, PVA/CS-2, PVA/CS-3), it can be seen that the PVA/CS-1 sample (containing PVA, CS, and TiO_2_ nanopowder) has the best antimicrobial activity against both Gram-positive bacteria (*Staphylococcus aureus*, MRSA) and Gram-negative bacteria (*Escherichia coli*, *Pseudomonas aeruginosa*). The yeast *Candida albicans* also proved to be sensitive to the action of the compounds in the PVA/CS-3 sample.

In the case of the PVA/CS-2 and PVA/CS-3 composite films containing both TiO_2_ nanoparticles and PFR-3 additive in their structure, the presence of TiO_2_ at a similar concentration as in PVA/CS-1 highlights the antimicrobial effect of the sample on all strains tested. The inclusion of PRF in their composition did not significantly alter the antimicrobial potential of the tested materials.

The tests performed in our study confirmed the well-recognized antimicrobial capability of TiO_2_. The antimicrobial potential and mechanisms of action of TiO_2_ on bacterial cells are complex and diverse, exhibiting a broad spectrum of antimicrobial activity against bacteria (Gram-positive and Gram-negative), yeasts, and implicitly antibiotic-resistant microorganisms [44]. The mechanism thought to be responsible for the antimicrobial effect of TiO_2_ is commonly associated with reactive oxygen species (ROS), which, when irradiated with bandgaps, produce photoinduced charges in the presence of O_2_ [45]. Thus, the oxidative effect alters several cellular structures, but the first to be affected are the cell wall and cell membranes, leading to cell lysis and loss of cell integrity [46]. Specific studies on antimicrobial mechanisms have shown that microorganisms exposed to photocatalytic TiO_2_ NPs exhibited cell inactivation at the level of the regulatory network and signal transduction, a significant reduction in respiratory chain activity, and inhibition of the ability to assimilate and transport iron and phosphorus. These processes, with the extensive cell wall and membrane changes, were the main factors explaining the biocidal activity of TiO_2_ NPs [44].

### 2.5. In Vitro Citotoxicity and Proliferation Assay

The cytotoxicity tests undertaken in this step of the study (MTT and cell morphology analysis) have a screening value and are performed and analyzed in accordance with ISO-10993-5 standards recommendations for cytotoxicity as a first-intended and eliminatory requirement for biocompatibility.

**Figure 7:**
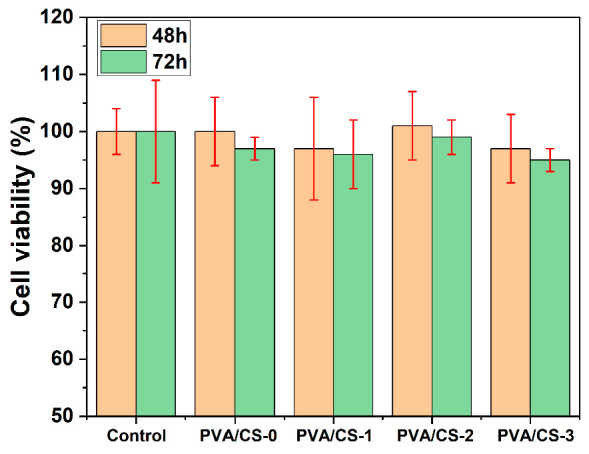
MTT test results for all PVA/CS composites.

PVA/CS composite films have been subjected to cytocompatibility tests that were performed by culturing MCF 7 in the physical presence of material samples (Figure 7). The MCF 7 cells are a standard line for cytocompatibility testing, as recommended by ASTM.

Figure 7 shows the results of the MTT test, in which cell viability was expressed as an average and standard variation, obtained from the processing of absorbance values. Cell viability in control cultures was considered 100% ± SD and experimental values—percentage of control value. Figure 7 shows the cell viability after 48 and 72 h of direct cell interaction with the material samples.

The test revealed that all analyzed material samples decrease the cell viability by about 20% compared to control cultures. On the other hand, cell viability in the experimental cultures is approximatively the same, regardless of the TiO_2_ ratio, which means that TiO_2_ does not influence the biocompatibility of the materials. The decreasing of the cell viability in experimental cultures with the same value could be explained through the mechanical action of the direct contact of the membranes on the cells. The in vitro experimental condition for cell viability testing leads to the conclusion that polyvinyl alcohol/chitosan matrices with TiO_2_ nanoparticles and/or polyphosphonate additive PFR-3 express a cytocompatibility, according to ISO 10993-5 recommendations.

### 2.6. Analysis of Cell Morphology

**Figure 8:**
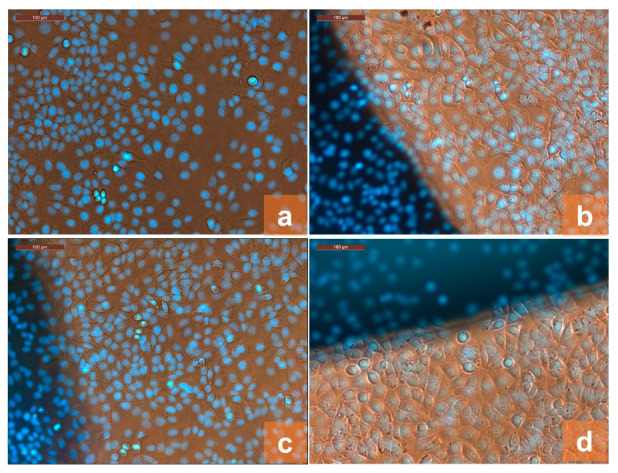
Microscopic images of DAPI-stained cells at 72 h of culturing in contact with PVA/CS-0 (**a**), PVA/CS-1 (**b**), PVA/CS-2 (**c**), and PVA/CS-0 (**d**) at 20× objective magnification.

The effect of direct exposure of cultured cells to the sample material was assessed microscopically by phase contrast, overlapped to fluorescence images of DAPI-stained nuclear DNA. The images of the fixed and stained cells at 20× objective magnification are shown in Figure 8.

From the images showing the experimental cultures, no differences of cell morphology were observed between the different compositions of the samples. All analyzed cultures contain epithelial cells with a shape typical of the MCF 7 cell line. It is observed that at 72 h of culture, the cells grow in confluent monolayers, with a cell density without significant differences between the binary PVA/CS sample and multi-component PVA/CS films containing TiO_2_ and/or PFR-3 additives.

## 3. Conclusions

PVA/CS composite films were obtained by the casting from solution technique, starting from appropriate amounts of PVA and CS, as polymer matrices, and TiO_2_ and PFR-3 polyphosphonate were utilized as reinforcing additives. Infrared spectroscopy enabled structural validation and the identification of distinctive bands for certain functionalities in the prepared composite films. According to the Ra value, the surface roughness characteristics decreased when the PFR-3 was introduced into the PVA/CH matrix, indicating its contribution to the homogeneity and quality of the four-component composites. Scanning electron microscopy demonstrated a small increase in porosity in the PVA/CS-3 sample as the polyphosphonate PFR-3 level increased. In vitro evaluation of antimicrobial potential in the series confirmed the antimicrobial role of TiO_2_ in the PVA/CS-TiO_2_ matrices. Cell viability testing revealed that polyvinyl alcohol/chitosan matrices containing TiO_2_ nanoparticles or TiO_2_/polyphosphonate additive PFR-3 exhibit cytocompatibility in accordance with ISO 10993-5 guidelines.

## 4. Materials and Methods

### 4.1. Materials

Partially hydrolyzed PVA (Mowiol 26–88 (KURARAY POVAL 26–88)) with a molecular weight of 160,000 g/mol and an 87.7 percent degree of hydrolysis was supplied by ZAUBA (Munchen, Germany) and used as received. Chitosan hydrochloride (Mw = 302.11 kDa, DAC degree = 82%), titanium (IV) oxide nanopowder (21 nm primary particle size), and glacial acetic acid were purchased from Sigma-Aldrich (St. Louis, MO, USA). 3,3′-Diaminodiphenyl sulfone, 4-hydroxybenzaldehyde, tetrahydrofuran anhydrous (THF), *N*,*N*-dimethylformamide (DMF), and phenylphosphonic dichloride were purchased from Sigma-Aldrich (Taufkirchen, Germany).

9,10-Dihydro-9-oxa-10-phosphaphenanthrene-10-oxide (DOPO) was purchased from Chemos GmbH, Germany and dehydrated before use. Polyphosphonate additive (PFR-3) has been prepared in our laboratory according to an adopted procedure from a previous work [47], starting from 3,3′-diaminodiphenyl sulfone and 4-hydroxybenzaldehyde. All other reagents were used as received from commercial sources.

### 4.2. Preparation of PFR-3 Polyphophonate Additive

In the first step, a DOPO-containing bisphenol was obtained via condensation reaction of 20 mmol 4-hydroxybenzaldehyde (2.44 g) with 10 mmol 3,3′-diaminodiphenyl sulfone, in 20 mL THF, followed by the in-situ addition of DOPO (40 mmol, 8.64 g solved in 15 mL THF) to the newly formed azomethine groups. PFR-3 have been prepared through polycondensation reaction of equimolecular amounts of phenylphosphonic dichloride and DOPO-containing bisphenol, in DMF.

PFR-3, Yield: 95%.

FTIR (KBr, cm^−1^): 3063 (=C–H), 1589 (C–C Aromatic), 1475 (P–Ar), 1353 and 1149 (O=S=O), 1200 (–P=O), 924 (P–O–Ar).

^1^H NMR (400 MHz, DMSO-d6, δ, ppm): 8.18–8.10 (m, 4H), 7.49 (m, 2H), 7.47–7.39 (m, 5H), 7.33–7.05 (m, 20H), 6.95–6.66 (m, 6H), 6.65–6.62 (m, 3H), 5.60–5.50 (m, 1H), 5.30–5.00 (m, 1H).

### 4.3. Preparation of PVA/CS Films following the Casting Procedure

**Figure 9:**
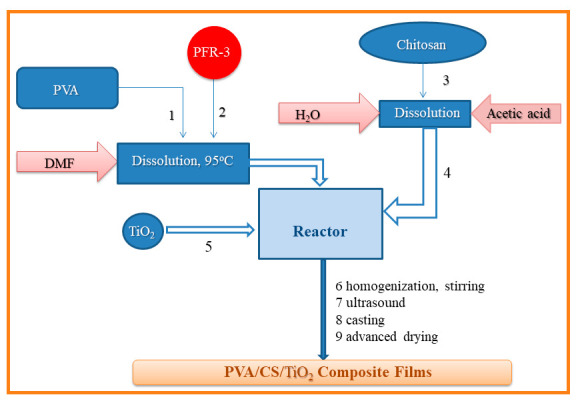
General scheme of the process for the preparation of films based on PVA and chitosan.

The first step in preparing these materials as composite films involved the treating of polyvinyl alcohol with oligophosphonate, a reaction which takes place in polar organic solvent, in this case DMF, working with concentrations of 5% PVA, by heating the mixtures at 95 °C for about 4–5 h. After the dissolution of the components is complete, the mixture is allowed to cool down to about 40 °C; then, the solution is poured into crystallizers, and the solvent is allowed to evaporate. The second stage consists in transferring the film obtained after drying in the crystallizer over a previously obtained chitosan solution (1% solution in water treated with glacial acetic acid 2%). After complete dissolution of the reaction mixture, the mixture was treated with an aqueous suspension of a predetermined amount of titanium dioxide nanoparticles, homogenized, and previously dispersed by ultrasound for 15 min. The resulting homogeneous composite mixture was poured into 10 × 10 cm^2^ Teflon plates to slowly evaporate the solvent in ambient conditions. Afterwards, they were placed in the oven for an advanced drying treatment by heating to approximately 40–50 °C, under a vacuum, for a period of 8 h. The obtained films are stored and used for the characterization and testing of new materials. The complete data on the synthesis of composites in this series are presented in Table 1, whereas the schematic diagram of the preparation is given in Figure 9.

### 4.4. Composite Films Structure and Performances Characterization

#### 4.4.1. Chemical Structure of PVA/CS/TiO_2_ Composite Films

The chemical structure of the PVA/CS/TiO_2_ films was analyzed using Bio-Rad ‘FTS 135′ FTIR spectrometer equipped with a Specac “Golden Gate” ATR accessory. To record scans between 4000 and 500 cm^−1^ at a resolution of 4 cm^−1^, a LUMOS Microscope Fourier Transform Infrared (FTIR) spectrophotometer (Bruker Optik GmbH, Ettlingen, Germany), equipped with an attenuated total reflection (ATR) device was used.

#### 4.4.2. Roughness and Contact Angle Measurements

The roughness of the composite films was analyzed at a recording speed of 0.10 mm/s using the Tencor Alpha-Step D-500 High Sensitivity Stylus Profiler (KLA-Tencor Corporation, Milpitas, CA, USA). By applying a stylus force of 15 mg and a long-range cutoff filter of 60 μm, arithmetic mean roughness, Ra, was obtained, which is the average of the absolute value along the sampling length.

Two test liquids—double-distilled water (W) and ethylene glycol (EG)—were used to test their static contact angles on the composite film surfaces. The measurements were performed in triplicate, at room temperature, on a CAM 101 system (KSV Instruments, Helsinki, Finland) equipped with a liquid dispenser, video camera, and drop shape analysis software. The geometric mean approach was employed to calculate the surface tension parameters. This measurement allows for the calculation of parameters that describe the composite film’s surface and its capacity for absorption, such as free surface energy (*SV*), solid-liquid interfacial tension (*SL*), and work of adhesion (*Wa*). Detailed experimental setup and calculations were described in a previous publication [34].

#### 4.4.3. Degree of Swelling

Swelling behavior of the PVA/CS composite films prepared via the solution casting method was studied. Thus, dried samples of 0.5 × 0.5 cm^2^ were weighted and submerged in 10 mL Millipore water in a closed bottle that was set in a thermostatic bath at 37 °C. The composite film samples were taken at predetermined intervals, and, after the extra water was removed with filter paper, the films weights were measured. The following equation was used to compute the swelling ratio, which represents the water absorption of each sample:

(1) $$SD\left(\%\right)=\frac{W_{t}-W_{d}}{W_{d}}\times 100$$

where SD (%) represents the amount of absorbed Millipore water; W_d_—weight of the dry composite film; W_t_—weight of the hydrated sample.

#### 4.4.4. Microscopic Morphology

Microscopic examinations have been performed on Environmental Scanning Electron Microscope Type Quanta 200, operating at 10 kV with secondary electrons in low vacuum mode (LFD detector). The composite samples were fractured, and their cross-section surfaces were analyzed using scanning electron microscopy (SEM). An Energy Dispersive X-ray (EDX) system is a feature of the Quanta 200 microscope that allows for qualitative, quantitative analysis, and elemental mapping.

#### 4.4.5. Evaluation of Antimicrobial Activity

Diffusimetric determinations were performed to highlight the antimicrobial activity of the PVA/CS samples containing PFR-3 and/or TiO_2_ additives. This technique is common for such tests and is based on the principle of contact of the test matrices with the surface of a culture medium inoculated with different microbial species. The antimicrobial tests were performed with standardized strains: *Staphylococcus aureus* ATCC 25923, *MRSA* ATCC 43300, *Escherichia coli* ATCC 25922, *Pseudomonas aeruginosa* ATCC 27853, and *Candida albicans* ATCC 90028. Standard microbial suspensions with a density of 0.5 McFarland, prepared with a spectrophotometer, were used. An aliquot (500 μL) of the bacterial suspension was applied to the surface of the Mueller-Hinton aggregate culture medium (BioRad, Taufkirchen, Germany) using an exudate swab. After drying for 10 min in a thermostat with the lid open, the test matrices were spread on the surface of the medium. The results were evaluated after incubation at 37 °C for 24 h. The antimicrobial activity was highlighted by identifying the areas of microbial inhibition formed upon contact with the tested matrices.

#### 4.4.6. Evaluation of Cytotoxicity and Cell Morphology

The PVA/CS composite films were cut into 4 mm Ø discs, decontaminated with 70% aqueous ethyl alcohol solution for 20 min and then repeatedly washed in sterile ultrapure water and HBSS (Hank’s Balanced Salt Solution) to remove any remaining contaminants. Following washing, the samples were equilibrated for 24 h in culture media (DMEM Ham/F12, supplemented with 10% fetal bovine serum and 1% antibiotic mixture). For equilibration, 0.5 mL of culture media were utilized for each piece of 4 mm Ø material. Using the MTT assay, the cytotoxicity of the growth medium and the material sample were both evaluated. Additionally, phase-contrast microscopy and fluorescence microscopy were used to examine the direct effects of the material samples on cell morphology. For biocompatibility study, the human epithelial cell line MCF 7, at the density of 20 × 10^4^/well cells, was plated in 48-well culture plates and DMEM Ham/F12 culture medium supplemented with 10% bovine fetal serum and 1% mixture of Penicillin-Streptomycin-Neomycin antibiotics (all for in vitro use). Thus, material samples or the media of their extraction were put over the cell monolayers in the 48-well culture plates in order to assess the viability, density, and morphology of the cells.

The MTT technique uses tetrazolium salts as an oxidized substrate for mitochondrial dehydrogenases to measure the activity of cellular metabolism. The basic idea behind the procedure is that the yellow MTT compound is reduced into an insoluble purple (formazan) product. The formazan salts are solubilized with isopropyl alcohol to obtain a blue-violet solution, the intensity of which is directly proportional to the number of living cells in the culture. Briefly, for MTT experiments, the culture medium of the cell cultures was replaced with work MTT solution of 0.25 mg/mL and incubated with cells for 3 h. The MTT solution was replaced with an equal volume of isopropyl alcohol to solubilize the formazan crystals. The absorbance of the formazan solution was measured spectrophotometrically at 570 nm, using a TECAN UV/VIS plate reader. An equivalent amount of isopropanol was used as the blank reference sample. The cell viability was calculate applying the equation:

(2) $$Cell\;viability\;\left(\%\right)=\frac{A_{Sample}}{A_{control}}\times 100$$

Each membrane sample was evaluated in triplicate, at 48 and 72 h of cell incubation with materials. The cultures developed in the absence of the material were used as growth control.

The cells were fixed in 4% paraformaldehyde solution and permeabilized for 30 min at +40 °C, in 0.05% Triton X 100 in PBS. After 72 h of interaction between the cells and material, the DAPI (2-(4-amidinophenyl)-1H-indole-6-carboxamidines) staining protocol was carried out for the cell morphology examination. PBS was used to properly clean the permeabilized cells before they were incubated with DAPI 10 μg/mL work solution for 30 min at room temperature. After being washed in PBS, stained cells were examined using fluorescence microscopy at λex 340 nm and λem 488 nm. To emphasize the cell nucleus and form, phase contrast was overlapped with the fluorescence.

## Figures and Tables

**Table 1:** Preparation details of the PVA/CS composite films.

Sample	PVA (g)	Chitosan (g)	PFR-3 (g)	TiO_2_ (g)
PVA/CS-0	1.5	0.5	-	-
PVA/CS-1	1.38	0.46	-	0.16
PVA/CS-2	1.2171	0.4057	0.2172	0.16
PVA/CS-3	1.0542	0.3514	0.4344	0.16

**Table 2:** Surface tension parameters of the test liquids used in contact angle measurements performed on PVA/CS composite films.

Sample	Roughness Ra (nm)	W_a_ (mN/m)	γ_SV_ (mN/m)	γ^P^ SV (mN/m)	γ^d^ SV (mN/m)	γ_SL_ (mN/m)
PVA/CS-0	570.3	65.24 (W) 62.38 (EG)	22.54	2.75	19.79	30.10
PVA/CS-1	208.1	67.65 (W) 77.08 (EG)	49.84	0.014	49.82	54.98
PVA/CS-2	78.6	75.76 (W) 77.16 (EG)	38.92	1.73	37.18	35.96
PVA/CS-3	65.5	65.54 (W) 61.84 (EG)	21.49	3.14	18.55	28.45

**Table 3:** Qualitative evaluation of the antimicrobial activity of APV-chitosan-PFR-TiO_2_ matrices.

Sample	*Staphylococcus aureus*	*MRSA*	*Escherichia* *coli*	*Pseudomonas aeruginosa*	*Candida albicans*
PVA/CS-0	+	+	+	+	+
PVA/CS-1	+++	++	++	+	++
PVA/CS-2	+	+	+	+	+
PVA/CS-3	+	+	+	+	+

Legend: [+] = inhibition zone present; [++] = clear inhibition zone the size of the matrix disc, [+++] = clear zone of inhibition went beyond the imprint area of the matrix disc.

## Data Availability

The data that support the findings of the current study are listed within the article.
